# Influence Analysis of Modified Polymers as a Marking Agent for Material Tracing during Cyclic Injection Molding

**DOI:** 10.3390/ma16186304

**Published:** 2023-09-20

**Authors:** Tom Eggers, Sonja Marit Blumberg, Frank von Lacroix, Werner Berlin, Klaus Dröder

**Affiliations:** 1 Volkswagen AG Wolfsburg, Berliner Ring 2, 38440 Wolfsburg, Germany; 2 Institute of Machine Tools and Production Technology, Technische Universität Braunschweig, Langer Kamp 19b, 38106 Braunschweig, Germany

**Keywords:** injection molding, tracing, traceability, polymers, marking agent, modified polymers, process optimization, process predictability, recycling, circular economy

## Abstract

Injection molding (IM) is already an established technology for manufacturing polymer products. However, in the course of the increased use of recyclates for economic and ecological reasons, its application capability has been confronted with new requirements for reliability and reproducibility. In addition, the IM process is confronted with regulations regarding a verifiable recycling degree in polymers. With regard to the material identification and storage of manufacturer-, process- or product-related data in polymers, the implementation of a material-inherent marking technology forms a potential answer. The IM process combined with modified polymers (MP) as a marking technology turns out to be a feasible approach to manufacturing reproducibly and offers a high quality based on increased process awareness and fulfilling the required traceability. Therefore, this work focuses on the trial evaluation of MP within the IM process. The influence of MP on the material process behavior and mechanical and thermal component properties, as well as the influence of the IM process and recycling on MP traceability, are investigated. No discernible influences of MP on the investigated properties could be identified, and the traceability from the initial material to a recyclate could be confirmed. MP is suitable for monitoring the aging state of polymers in IM.

## 1. Introduction

Injection molding (IM) is among the most prominent representatives of polymer-based manufacturing processes and is characterized by a causal flow from the initial material to the end product. In the IM process, the plastification of granular or powdered polymers into a flowable state occurs using dissipation and heat conduction along a temperature-controlled cylinder. The plastified material is injected into the closed mold via the axial movement of the injection unit and subsequently solidifies. In the case of thermoplastic materials, the mold is temperature-controlled to a temperature below the solidification temperature of the melt so that the melt cools and solidifies as it enters the mold. Part quality is influenced by the IM process parameters, such as the temperatures from the hopper to the nozzle, the holding pressure and the back pressure, as well as the screw rotation speed, holding time and mold temperature [1,2,3,4].

The widespread use of thermoplastics and their composites in the present is a cause of growing concern because of their adverse effects on the environment [5]. Although the use of recyclates in the manufacturing of new polymer products is an important contribution to a circular economy, as it enables material cycles to be closed, the amount of recyclates in new products is still low due to technical barriers to reprocessing [6]. The remaining fraction ends up in landfills or in the oceans and becomes microplastics [7,8]. According to Yang et al. [9], a current barrier to the use of recycled polymers is the lower quality of recyclates compared to virgin materials. Even though mechanical recycling is less energy-intensive than chemical recycling [10], it is capable of having a significant impact on the quality of the recyclates [11,12]. The material is subject to various material aging processes [13,14,15]. Besides the rheological properties [16,17,18], the mechanical properties are also affected due to the reduction in molecular weight [10,11,18,19,20,21,22,23,24,25,26,27,28,29]. As a result, the recycling of polymers tends to decrease the stability of the IM process and decrease part quality [14,21,30]. A common method of reusing recycled polymers is to mix them with virgin materials to obtain materials with average properties [21,31,32,33,34,35,36]. A ratio of approximately 30% [21] to 50% [19] recycled to virgin material was reported in previous studies.

As different batches and material streams have different material properties, the identification of the different classes of recyclates is required [5,9,32,36,37]. In Auer et al. [37], the current problems of recycling and their causes were summarized. Although the use of methods to determine material composition is a solution to reduce quality problems, Auer et al. [37] evaluated the current sorting technology as inappropriate. For this, Auer et al. [37] mentioned the use of tracing technologies that can sort the previously unsortable materials. Modified polymers were evaluated as a suitable material-inherent tracing technology for use in polymer processing manufacturing [38]. This tracing technology has already been successfully investigated in the selective laser sintering of polyamide 12 in a previous study [39]. Modified polymers are sequence-defined polymers, and they are encodable via targeted polymerization so that information can be stored at the molecular level [40].

IM combined with modified polymers as a marking technology turns out to be a promising way to manufacture reproducibly and with high quality, based on an advanced process awareness [41]. Therefore, it is based on the concept of the tracer-based optimization of the process [38] via a material marking. Material traceability in injection molding is achievable due to the incremental addition of one or more different modified polymers at each new recycling step the material passes through. The markers are analyzed at defined analysis points in the recycling process and before the IM process. It is intended to determine the mixture of the material on the basis not only of either the qualitative or the quantitative analysis of the markers but also via a combination of both variants. Thus, an increasing amount of different modified polymers propagates through the continuous material in a manner analogous to the recyclate classes formed. The composition of the markers can, in turn, be used to infer the different recyclate proportions. Once the history of the material or its composition is identified, it is possible to draw conclusions about the quality of the material. The respective marker codes are linked to the material history in a database, which is expanded via new codes with each new recycling of the material. If the recyclate proportions are known, an overall material quality can be defined from the respective material qualities of the individual recyclate proportions, and the process parameters can be specifically adjusted, as a result of which the scattering material properties are compensated for. As the material is refreshed via virgin material, the proportions of the frequently recycled materials, as well as the associated markers, decrease. When the respective marker concentration falls below a certain limit, traceability is no longer guaranteed. The associated amount of recyclate is then already so small that it can be neglected. In addition to the stepwise material marking, the mechanical properties of marked products must also be examined to maintain the functionality of products.

Consequently, the aim of the present study is to validate modified polymers for application in IM. Investigations are carried out to determine the influence of the modified polymer on the processing behavior of the material and the component properties and to determine the influence of the IM process and recycling on the traceability of the modified polymer. Thermal and rheological material properties, as well as mechanical and thermal component properties, are analyzed. Since repeated extrusion and IM are evaluated as suitable methods to investigate recyclability [29,42,43,44,45,46], these methods are used in the present study. The influence of the cyclic processing of the marked material, in particular, thermal stress and stress due to shear forces [1,2,3,4,47], on the functionality and detectability of the marker is investigated. Mass spectroscopy is used to determine the traceability of the marker.

## 2. Materials and Methods

In order to minimize disturbance variables and avoid aging processes due to humidity, temperature and ultraviolet radiation [13,14,21,48], materials and test specimens were stored, and test procedures were applied under controlled ambient conditions of 23 °C and 50% relative humidity. The storage of materials and test specimens was airtight and protected from ultraviolet radiation.

### 2.1. Injection Molding Material

The Daplen EF155AE elastomer-modified polypropylene compound with 10% mineral filling from Borealis AG (Vienna, Austria) was used. This material is among the thermoplastic polyolefins [13,15,49] and can be easily processed with commercially available injection molding machines. Pre-drying the material at 80 °C for approximately 2 h was recommended in order to avoid residual moisture. Referring to the manufacturer’s data, the specific material density was 0.95 g/cm^3^. The material choice was based on the fact that polypropylene is one of the most commonly used semi-crystalline thermoplastics in injection molding [13,50,51,52] and the most widely used polymer in the automotive industry [53].

### 2.2. Modified Polymer

The modified polymer used in this study was supplied by Polysecure GmbH (Freiburg, Germany) and branded as POLTAG^®^ technology [41,54,55,56]. A previous study [38] listed further information about the marking agent. Compared to the investigated modified polymer used in a previous study [39], this modified polymer contained a different main molar mass, as well as different mass numbers of the sequences (Table 1).

### 2.3. Production of the Master Batch

On the basis of the used injection molding material and modified polymer, a master batch was produced. Via a spray-drying process, the modified polymer was added to the injection molding material via mixing. In the master batch, a concentration of 30 ppm of the modified polymer was used. The method of producing the master batch based on the modified polymer was confirmed in a previous study [39]. Through weighing the material fractions, the concentrations of the modified polymer in the material were determined to be 1 ppm and 10 ppm. For the quantification of the material mass, an EW 4200-2NM scale from KERN & Sohn GmbH (Balingen-Frommern, Germany) was applied.

### 2.4. Injection Molding Processing and Recycling Procedure

**Figure 1:**
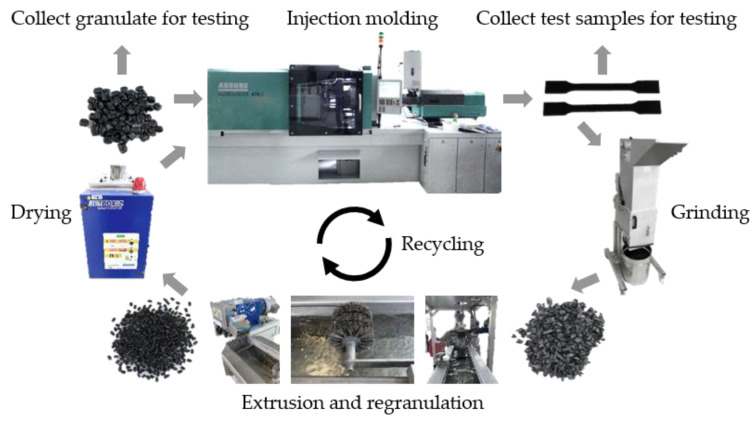
Process flow of the used recycling steps to process of the used injection molding material. The process flow was divided into injection molding, collecting of the specimens for testing (tensile bars), grinding of the remaining specimens, extruding and regranulation of the shredded material, drying of the granulate, collecting of the granulate for testing and renewed injection molding.

In Figure 1, the injection molding and mechanical recycling process used are illustrated. The presented process flow of recycling was in accordance with Tamrakar et al. [20]. A horizontal injection molding machine [1] of the Allrounder 470 E Golden Electric type from ARBURG GmbH + Co KG (Loßburg, Germany) was used for injection molding. In the initial process cycle (R0), unmarked and marked granulates were fed into the injection molding machine to produce test samples. All samples were produced via injection molding using the parameters listed in Table 2. The parameters were in accordance with the DIN EN ISO 19069-2:2020-01 [57] standard, the parameters listed in Isayev et al. [1] and Dominghaus et al. [51] and the specifications of the manufacturer of the injection molding machine. The cylinder temperature was gradually increased from the hopper to the nozzle end to ensure a smooth transition from a solid to a molten polymer and to reduce the screw wear [1,2,20,47]. A defined number of specimens were taken for further characterization.

In Table 3, the investigated samples of each injection molding process are listed. The rest of the test samples were ground using a granulator of the C17.26sv SE type from Wanner Technik GmbH (Wertheim-Reicholzheim, Germany) and a grinding sieve with a mesh size of 6 mm.

Then, the ground material was extruded by a twin screw extruder of the ZE25A-40D-UTX-UG type from KraussMaffei Berstorff (Laatzen, Germany) with gravimetric dosing from Scholz Dosiertechnik GmbH (Großostheim, Germany). All materials were extruded according to the parameters listed in Table 4. The extruded strands were cooled in a water bath using a strand pelletizing unit of the ips-SG-E 30 Kombi type from IPS Intelligent Pelletizing Solutions GmbH & Co KG (Niedernberg, Germany) with an integrated strand cooling tank, dried via blasting with an integrated strand dewatering system and processed to a granulate with a granulate size of 3 mm. A cold cutting process [1,47] was applied in this case. Next, the granulates were dried at 80 °C for a duration of 2 h in a JETBOXX granulate dryer from HELIOS GmbH (Rosenheim, Germany).

Before the next cycle (R1), a granulate quantity of 20 g was taken for further tests. The remaining amount of granulates was further processed. Then, the pre-dried granulates were injection-molded, and the recycling process was repeated up to ten times (R10). The number of recycling steps was based on Aurrekoetxea et al. [29], and it indicated an asymptotic behavior of the investigated thermal and mechanical properties after ten recycling steps. For each investigated material, an initial amount of 5 kg of the respective material was provided. Before each cycling step, the hopper, screw and mold were cleaned using compressed air and a vacuum cleaner, as well as dry wipes.

### 2.5. Thermogravimetric Analysis

Thermogravimetric analysis (TGA) was applied using a TGA/DSC-1 measuring system from Mettler Toledo (Gießen, Germany). Measurements were performed according to the DIN EN ISO 11358-1:2022-07 [59] standard. The change in the mass of a sample over a defined, time-related temperature curve under the influence of a purge gas was determined. Whether marking with the used modified polymer led to a different thermally induced degradation behavior of the polymer was examined [13,60]. The sample was heated from room temperature to 900 °C at a heating rate of 20 K/min under a nitrogen atmosphere (flow: 70 mL/min) and was stored at this temperature and atmosphere for 10 min. Afterwards, the sample was cooled to 400 °C at a cooling rate of 20 K/min under a nitrogen atmosphere (flow: 70 mL/min). Next, the sample was heated to 900 °C under the presence of oxygen (flow: 70 mL/min) at a heating rate of 20 K/min and was stored at this temperature and atmosphere for 15 min. The sample weight for each measurement was 12 mg to 14 mg. The analyzed TGA measurement results included the maximum pyrolytic degradation temperature and the residue as mean values. No standard deviation was provided by the TGA measurement.

### 2.6. Differential Scanning Calorimetry Testing

Differential scanning calorimetry (DSC) was performed using a DSC-3^+^ measuring system from Mettler Toledo (Gießen, Germany). Measurements were obtained according to the DIN EN ISO 11357-1:2023-06 [61] standard and were realized under the presence of a nitrogen atmosphere. Each measurement was carried out using a sample weight of 10 mg ± 2 mg. The heating and cooling cycles were performed in the temperature interval between 50 °C and 200 °C. The temperature rate was 10 °C/min. Among other data from the DSC measurement, both the melting and crystallization temperatures and the onset temperatures were output as mean values. Whether marking with the used modified polymer tightened or widened the thermal process window of the polymer was considered. The DSC testing did not provide any standard deviation.

### 2.7. Melt Flow Index Test

Melt flow index testing was realized with an Mflow measuring device from Zwick/Roell GmbH & Co. KG (Ulm, Germany) and was performed according to the DIN EN ISO 1133-1:2012-03 [62] standard. In order to determine the melt flow rate (MFR), a testing load of 2.16 kg at a temperature of 230 °C and a filling quantity of 4 g was used. Melt flow index measurement is a single-point test method that represents a single measuring point of the viscosity curve and gives an initial indication of a change in the rheological behavior of the melt [63]. Whether the modified polymer used had an influence on the MFR value of the polymer was considered. To avoid moisture, the material was pre-dried at 80 °C for 2 h using a JETBOXX granulate dryer from HELIOS GmbH. Before testing, the nozzle was cleaned with cleaning tools and cotton cloths. The test started at a position of the piston of 50 mm. Thereby, five sections with a measured length of 5 mm were recorded. The mass of each of the five sections was quantified using an AB-100 scale from PCE Deutschland GmbH (Meschede, Germany). The extruded mass within the defined interval described the MFR value, which was expressed in the unit g/10 min.

### 2.8. Tensile Test

A tensile test was performed on a tensile testing machine of the Zwick/Roell Z100 type from Zwick/Roell GmbH&Co. KG. The equipment of the testing machine included a makroXtens mechanical extensometer and two wedge clamping jaws designed for a normal force of up to 10 kN. Furthermore, the equipment included an Xforce K load cell determined for the same load limit and a testControl II control unit. According to the DIN EN ISO 19069-2:2020-01 [57] standard, the tensile test specimens (Table 3) were conditioned in a constant climate chamber of the KBF 240 type from BINDER GmbH (Tuttlingen, Germany) for 96 h at a temperature of 23 °C and 50% relative humidity. During the transfer from the constant climate chamber to the tensile testing machine, the samples were stored in airtight containers together with silicate pellets for moisture absorption. Tensile testing was performed from the conditioned state of the samples and was done within ten days after manufacturing the samples. The test specimens were evaluated according to the DIN EN ISO 527-1 [64] standard. According to the DIN EN ISO 19069-2:2020-01 [57] standard, the tensile strength and elongation at break were measured at a test speed of 50 mm/min. In contrast, Young’s modulus was measured at a speed of 1 mm/min. In addition, Young’s modulus was determined as the secant modulus, according to the DIN EN ISO 527-1 [64] standard, in the interval of elongation from 0.05% to 0.25%. Thereby, the specimens were exposed to a preload of 0.1 MPa at a test speed of 1 mm/min. Five specimens of each process step were tested, and the mean value, as well as the standard deviation, was reported.

### 2.9. Charpy Impact Test

A Charpy impact test was performed on a pendulum impact tester of the Zwick/Roell HIT25P type from Zwick/Roell GmbH & Co. KG using a testControl II control unit. According to the DIN EN ISO 179-1:2010-11 [65] standard, the test specimens for Charpy impact testing were conditioned in a constant climate chamber of the KBF 240 type from BINDER GmbH for 96 h at a temperature of 23 °C and 50% relative humidity. For Charpy impact testing, a test specimen of type 1 was used. Due to the toughness of the material used, a Charpy impact test was performed according to the DIN EN ISO 179-1/1fA [65] standard. The specimens were prepared from the parallel part of the specimens presented in Table 3 and corresponded to the multipurpose test for specimen type A, according to the DIN EN ISO 3167:2014-11 [66] standard. During the transfer from the constant climate chamber to the pendulum impact tester, the specimens were stored in airtight containers together with silicate pellets for moisture absorption. The Charpy impact test was performed with the conditioned state of the samples and was done within ten days after manufacturing the samples. A 7.5 J pendulum was used in this test. As a result of the Charpy impact test, among other data, Charpy double-edge-notched impact strength (dNIS) was analyzed. Ten specimens of each process step were tested, and the mean value, as well as the standard deviation, was reported.

### 2.10. Vicat Softening Temperature Test

The Vicat softening temperature (VST) of the specimens was measured according to the DIN EN ISO 306:2023-03 [67] standard using a VST/HDT Compact 3 system from Coesfeld GmbH & Co. KG (Dortmund, Germany). The system used consisted of a bar with a support disk for the test weights and a fixture for the indenter tip, as well as a calibrated dial gauge for determining the indentation depth. Before the measurement, the surface of the specimen was cleaned of burrs and irregularities. Based on the B 50 VST method, the specimens were loaded with a force of 50 N, and the heating rate was 50 °C/h. During measurement, the specimens were positioned on a specimen fixture and heated at the defined heating rate in a silicone bath. The specimens were prepared from the end pieces of the specimens presented in Table 3, and the size of the specimens was 10 mm × 10 mm × 4 mm. The temperature at which a tip penetrated 1 mm deep into the surface of the test specimen was determined. The indenter tip has a circular cross section of 1 mm^2^. The measured temperature was defined as the VST. Three specimens were used for each measurement. The mean value and the standard deviation were used to determine the VST. The objective is to quantitatively characterize the thermal stability of a polymer [68]. Since the VST responds to a change in molecular size, the measurement can be used to indicate the processing-induced thermal damage of the material [68,69].

### 2.11. Tandem Mass Spectroscopy

Tandem mass spectroscopy (MS/MS) was used to detect, sequence and measure the traceability of the modified polymer [40,70,71,72]. An MS/MS device of the API 4000 MS/MS type was used, which was supplied by AB SCIEX (Darmstadt, Germany). The method for detection, sequencing and determining the traceability of the modified polymer was analogous to that of a previous study [39]. With the exception of the extraction methods listed in Table 5, the same procedure and parameters were used. The extraction methods depended on the investigated specimens. According to a previous study [39], the analyte was delivered to the atmospheric pressure chemical ionization source using a syringe pump of the Model 100 type from kd Scientific Inc. (Holliston, MA, USA). The syringe pump flow rate was 0.06 mL/h [39]. Detection and sequencing were both recorded for at least 5 min. Between each measurement, the MS/MS device was cleaned. Therefore, a liquid consisting of methanol and 3 mmol ammonium acetate was injected for at least 10 min.

Based on a Python tool used in a previous study [39], the results of the MS/MS measurements were evaluated. For this purpose, the sum of the determined intensities within a measurement interval of ±1 Da was determined for all mass numbers of the modified polymer (Table 1). The respective maximum peak of the mass spectrum was used to calibrate the molar mass. The modified polymer was considered to be detected if an intensity greater than 0 was determined for the mass number of the main mass, as well as for the majority of the mass number of all sequences within the measurement interval, and peaks were clearly visible [39].

## 3. Results

### 3.1. Influence of the Modified Polymer Used on the Material Properties

#### 3.1.1. Initial Material Properties

In Table 6, the investigated initial material properties for the injection molding material and master batch are presented. The determined crystallization and melting temperatures, as well as their onset temperatures, deviated between the injection molding material and the master batch by less than approximately 1%. Regarding the thermogravimetric analysis, the master batch shows an approximately 0.34% higher maximum pyrolytic degradation temperature and an approximately 0.55% higher residue than the injection molding material. The values of the melt flow rate (MFR) differ by approximately 2.4%. Considering the standard deviation, the deviation of the MFR values is approximately 0.1%.

#### 3.1.2. Thermal Process Window

**Figure 2:**
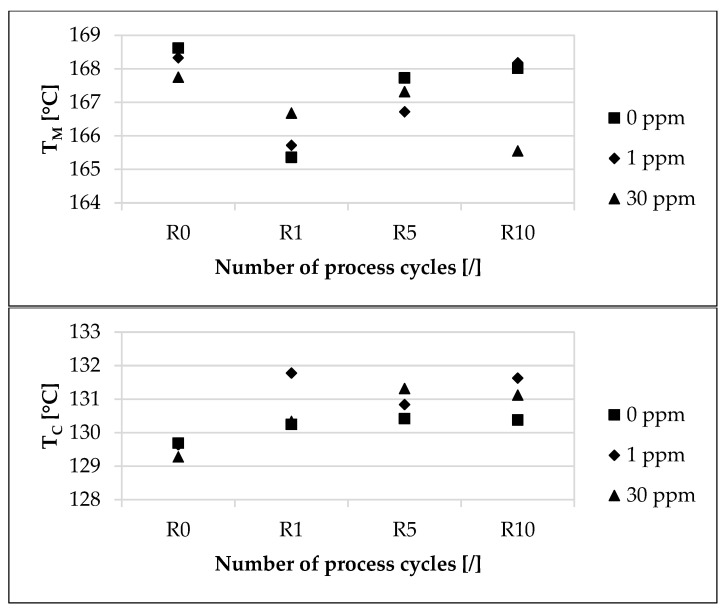
Influence of the modified polymer used and the recycling on the melting (T_M_) and crystallization temperature (T_C_). The material samples are based on the polypropylene compound injection molding material used and were collected in a dry state before each process step. Concentrations of the modified polymer used of 0 ppm, 1 ppm and 30 ppm were studied. The recycling steps are listed on the X-axis. Process step R0 describes the initial injection molding process step. Steps R1, R5 and R10 describe the first, fifth and tenth process steps of the recycling.

The influence of the modified polymer on the thermal process window, characterized by melting and crystallization temperature and depending on the concentration of the modified polymer and the recycling step, is presented in Figure 2. While there was an increase in melting temperature (T_M_) between the first and fifth steps for all concentrations considered, there was a decrease in T_M_ from R0 to R10 for all concentrations observed. From R0 to R10, the T_M_ for 0 ppm decreased by approximately 0.36%. In contrast, for 1 ppm, the T_M_ decreased by approximately 0.09% from R0 to R10, and for 30 ppm, it decreased by approximately 1.31%. At R0, the deviations of the T_M_ between the unmarked material and the marked materials amounted to a maximum of approximately 0.34%. At R1, the maximum deviation of T_M_ was approximately 0.8%. At R10, the deviations of the T_M_ between the unmarked material and the marked materials amounted to a maximum of approximately 1.47%.

Regarding crystallization temperature (T_C_), there was an increase from R0 to R10 for nearly all concentrations considered. From R0 to R10, the T_C_ for 0 ppm increased by approximately 0.53%. For 1 ppm, the T_C_ increased by approximately 1.54% from R0 to R10, and for 30 ppm, it increased by approximately 1.42%. At R0, the deviations of the T_C_ between the unmarked material and the marked materials amounted to a maximum of approximately 0.32%. At R1, the maximum deviation of T_C_ was approximately 1.17%. At R10, the deviations of the T_C_ between the unmarked material and the marked materials amounted to a maximum of approximately 0.96%.

#### 3.1.3. Melt Flow Rate

**Figure 3:**
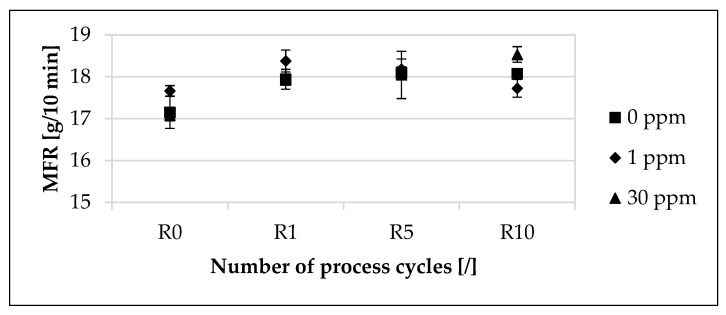
Influence of the modified polymer used and the recycling on the melt flow rate (MFR). The material samples are based on the polypropylene compound injection molding material used and were collected in a dry state before each process step. Concentrations of the modified polymer used of 0 ppm, 1 ppm and 30 ppm were studied. The recycling steps are listed on the X-axis. Process step R0 describes the initial injection molding process step. Steps R1, R5 and R10 describe the first, fifth and tenth process steps of the recycling.

The influence of the modified polymer on the mean value of the melt flow rate (MFR), depending on the concentration of the modified polymer and the recycling step, is presented in Figure 3. While the mean value of the MFR decreased for individual concentrations of the respective process cycles, the mean value of the MFR increased in the overall view from R0 to R10.

The mean value of the MFR for 0 ppm increased by approximately 4.61% from R0 to R1. In contrast, for 1 ppm, the mean value of the MFR increased by approximately 4.08% from R0 to R1, and for 30 ppm, it increased by approximately 4.92%. From R1 to R10, the mean value of the MFR for 0 ppm increased by approximately 0.72%. In contrast, for 1 ppm, the mean value of the MFR decreased by approximately 3.43% from R0 to R10, and for 30 ppm, it increased by approximately 3.40%. At R0, the deviations of the mean value of the MFR between the unmarked material and the marked materials amounted to a maximum of approximately 2.97%. At R1, the maximum deviation of the mean value of the MFR was approximately 2.45%. At R10, the deviations of the mean value of the MFR between the unmarked material and the marked materials amounted to a maximum of approximately 2.55%. The deviations of the mean values of the MFR as a function of the concentration of the modified polymer were also within the standard deviation across the recycling steps.

### 3.2. Influence of the Modified Polymer Used on the Component Properties

#### 3.2.1. Tensile Test

**Figure 4:**
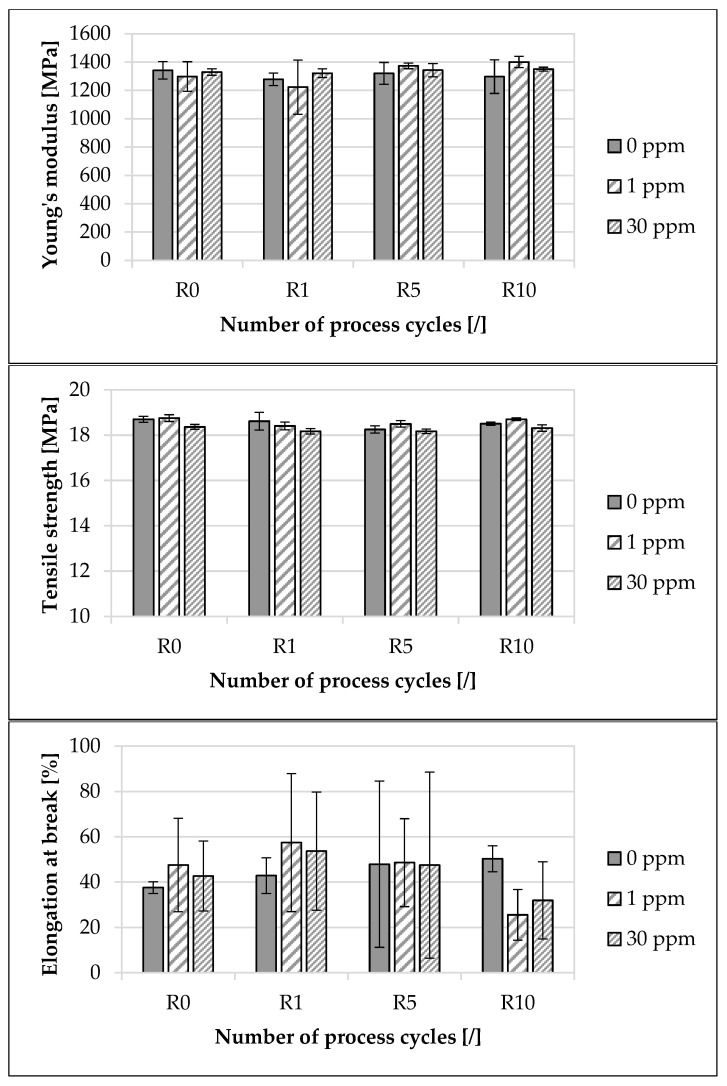
Influence of the modified polymer used and the recycling on the investigated mechanical properties. The specimens were manufactured from the polypropylene compound injection molding material used. Concentrations of the modified polymer used of 0 ppm, 1 ppm and 30 ppm were examined. The recycling steps are listed on the X-axis. Process step R0 describes the initial injection molding process step. Steps R1, R5 and R10 describe the first, fifth and tenth process steps of the recycling. In each case, five tensile bars were analyzed.

The influence of the modified polymer used on the mechanical properties, depending on the concentration of the modified polymer and the recycling step, is presented in Figure 4. With regard to the mean value of Young’s modulus for 0 ppm, there was a decrease of approximately 3.3% from R0 to R10. In contrast, for 1 ppm, from R0 to R10, an increase of approximately 7.92% was recorded, and for 30 ppm, from R0 to R10, an increase of approximately 1.59% was recorded. With regard to the mean value of Young’s modulus at R0, the deviation between 0 ppm and 30 ppm was approximately 0.93%. At R10, this deviation amounted to approximately 4.08%. With regard to the mean value of the tensile strength for 0 ppm, there was a decrease of approximately 1.02% from R0 to R10. In contrast, for 1 ppm, from R0 to R10, a decrease of approximately 0.32% was recorded, and for 30 ppm, from R0 to R10, a decrease of approximately 0.33% was recorded. With regard to the mean value of the tensile strength at R0, the deviation between 0 ppm and 30 ppm was approximately 1.76%. At R10, this deviation amounted to approximately 1.03%. Regarding the mean value of elongation at break for 0 ppm, there was an increase of approximately 34% from R0 to R10. In contrast, for 1 ppm, from R0 to R10, a decrease of approximately 46.2% was recorded, and for 30 ppm, from R0 to R10, a decrease of approximately 25.1% was recorded. With regard to the mean value of elongation at break at R0, the deviation between 0 ppm and 30 ppm was approximately 13.6%. At R10, this deviation amounted to approximately 36.5%. The deviations of the mean values of Young’s modulus, the tensile strength and the elongation at break, depending on the concentration of the modified polymer, were within the standard deviation even across the recycling steps.

#### 3.2.2. Charpy Impact Test

**Figure 5:**
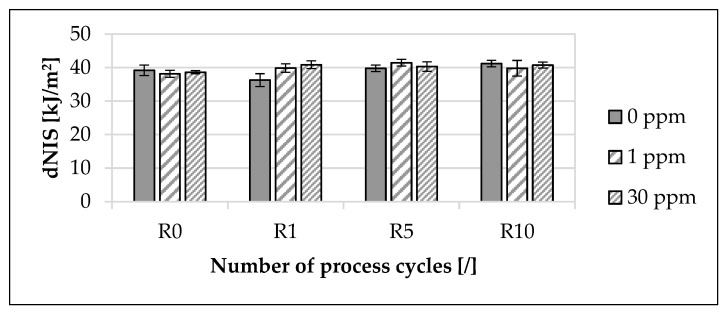
Influence of the modified polymer used and the recycling on the double-edge-notched impact strength (dNIS). The specimens were manufactured from the polypropylene compound injection molding material used. Concentrations of the modified polymer used of 0 ppm, 1 ppm and 30 ppm were examined. The recycling steps are listed on the X-axis. Process step R0 describes the initial injection molding process step. Steps R1, R5 and R10 describe the first, fifth and tenth process steps of the recycling. In each case, ten specimens were analyzed.

The influence of the modified polymer on the double-edge-notched impact strength (dNIS), depending on the concentration of the modified polymer and the recycling step, is presented in Figure 5.

The mean value of the dNIS for 0 ppm increased by approximately 5.13% from R0 to R10. In contrast, for 1 ppm, the mean value of the dNIS increased by approximately 4.33% from R0 to R10, and for 30 ppm, it increased by approximately 5.52%. At R0, the maximum deviation of the mean value of the dNIS between the unmarked material and marked materials was approximately 2.63%. At R10, the maximum deviation of the mean value of the dNIS between the unmarked material and marked materials was approximately 3.38%. The deviations of the mean values of the dNIS, depending on the concentration of the modified polymer, were within the standard deviation even across the recycling steps, with the exception of recycling step R1.

#### 3.2.3. Vicat Softening Temperature

**Figure 6:**
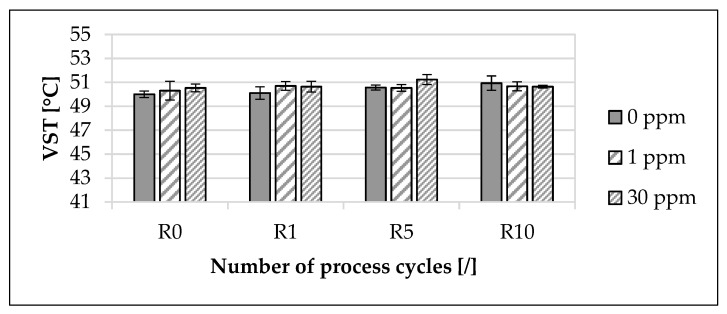
Influence of the modified polymer used and the recycling on the Vicat softening temperature (VST). The specimens were manufactured from the polypropylene compound injection molding material used. Concentrations of the modified polymer used of 0 ppm, 1 ppm and 30 ppm were examined. The recycling steps are listed on the X-axis. Process step R0 describes the initial injection molding process step. Steps R1, R5 and R10 describe the first, fifth and tenth process steps of the recycling. In each case, ten specimens were analyzed.

The influence of the modified polymer on the Vicat softening temperature (VST), depending on the concentration of the modified polymer and the recycling step, is presented in Figure 6. The mean value of the VST for 0 ppm increased by approximately 1.9% from R0 to R10. In contrast, for 1 ppm, the mean value of VST increased by approximately 0.74% from R0 to R10, and for 30 ppm, it increased by approximately 0.2%.

The maximum deviation of the mean value of the VST between the unmarked material and marked materials at R0 was approximately 1.06%. At R10, the maximum deviation of the mean value of the VST between the unmarked material and marked materials was approximately 0.59%. The deviations of the mean values of the VST as a function of the concentration of the modified polymer were also within the standard deviation across the recycling steps.

### 3.3. Traceability of the Modified Polymer Used

**Figure 7:**
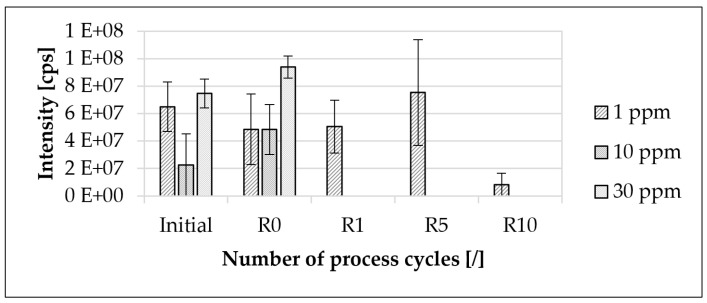
Traceability of the modified polymer used at different process steps. The recycling steps are listed on the X-axis. The initial step describes the initial granulate before injection molding processing. Process step R0 describes the initial injection molding process step. Steps R1, R5 and R10 describe the first, fifth and tenth process steps of the recycling. The specimens are based on the polypropylene injection molding material used. The arithmetic mean of the sum of the intensities within the considered interval of ±1 Da around the main molar mass of three MS/MS measurements each was formed. For the initial step and step R0, concentrations of the modified polymer used of 1 ppm, 10 ppm and 30 ppm were considered. For steps R1, R5 and R10, a concentration of 1 ppm of the modified polymer used was assumed.

**Figure 8:**
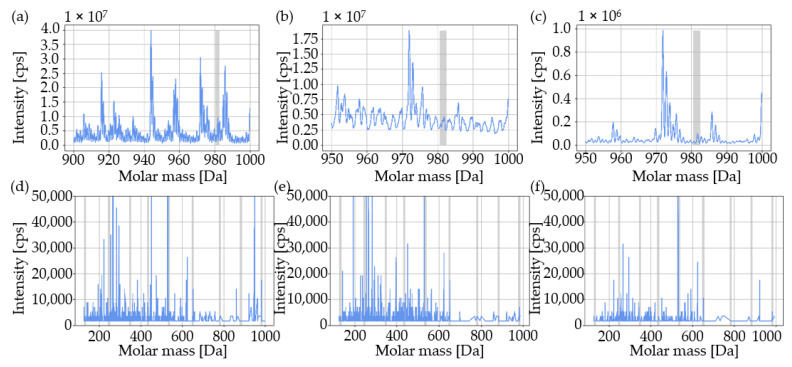
MS/MS measurements (detection and sequencing) of the selected process steps for the concentration of the modified polymer used of 1 ppm. The intensity is plotted over the molar mass: (**a**,**d**) initial injection molding material; (**b**,**e**) initial injection molding process step R0; and (**c**,**f**) tenth process step R10 of recycling. The intervals in which the main masses and individual sequences were detected are highlighted with gray bars. The specimens are based on the polypropylene injection molding material used.

The traceability of the modified polymer at different recycling steps is presented in Figure 7. The modified polymer was detectable at all investigated concentrations down to a concentration of 1 ppm, and the main mass, as well as all individual sequences, was detectable in all recycling steps (Figure 8).

The determined intensities did not increase linearly with the selected concentrations. Furthermore, there was no apparent correlation between the concentration of the modified polymer and the mean value of the detected intensity of the main mass (Figure 7 and Figure 8). If the standard deviation is taken into account, there was no change in intensity as a result of the initial injection molding process for all the investigated concentrations of the modified polymer used. In the initial material, from 1 ppm to 30 ppm, there was an increase in the mean value of the intensity of the modified polymer of approximately 14.6%. In contrast, the mean value of the intensity of the modified polymer decreased from 1 ppm to 10 ppm by approximately 65.2%. From the initial material to R0, the mean value of the intensity of the modified polymer used at a concentration of 1 ppm decreased by approximately 25.4%. In contrast, the mean value of the intensity of the modified polymer at 30 ppm increased by approximately 25.9%. For R0, the deviation between 1 ppm and 30 ppm relative to the mean value of the intensity of the modified polymer used was about 93.6%. From the initial material to R5, the mean value of the intensity of the modified polymer at 1 ppm increased by about 15.9%. In contrast, the average intensity of the modified polymer at 1 ppm decreased by 89.2% from R5 to R10. At R10, eight out of nine sequences were detectable.

## 4. Discussion

The investigated thermal and rheological material properties (Table 6, Figure 2 and Figure 3), as well as the mechanical (Figure 4 and Figure 5) and thermal component properties (Figure 6), were not significantly influenced by the modified polymer. On the one hand, this observation is confirmed by the marginal deviations of the mean values of the respective properties with increasing concentrations. If the minimum and maximum standard deviations are taken into account, the variation in the respective properties due to the presence of the modified polymer used was negligible. This observation can be attributed to the selected concentrations of the modified polymer used. These were too low to identify any influence on the investigated material and component properties via the chosen testing methods [39]. On the other hand, this observation is confirmed by a previous study [39] that investigated modified polymers as marking agents in a polyamide 12 material via selective laser sintering. Taking into account the concentrations of the modified polymer used in the base material used and the selected scope of the investigation, no discernible influence on the material and component properties was observed. Since comparable material and component properties, as well as comparable concentrations of the marking agent, were used in this study, the influence of the marking agent on the material and component properties identified in the present study was also evaluated as not discernible. As a result, the processing properties of the marked material in injection molding remained the same as those of the unmarked material. This is confirmed by the fact that the chosen concentration of 30 ppm in the master batch was already at the maximum and, thus, lower concentrations behave analogously in the investigated material [39]. Furthermore, any influence attributable to the modified polymer could also be masked by other additives in the investigated material [13,24,36,73,74,75,76,77,78,79,80,81].

The selected concentrations of the marking agent are in accordance with an earlier study [39]. In addition to the easier master batch handling to set lower concentrations, the choice was based on the use of minimal concentrations for a future marking agent application in injection molding. Furthermore, the concentration of the master batch was selected in consultation with the manufacturer of the modified polymer. With regard to possible stepwise material marking and process optimization [38], at an initial concentration of the modified polymer of 1 ppm per process cycle, a maximum concentration of no more than 30 ppm can be expected after 30 process cycles. This total results from the stepwise addition of modified polymers. Due to the usual recycling rates in injection molding [19,21], the respective concentration of the specific modified polymer (initially 1 ppm) after more than 30 process cycles is so low that this concentration, as well as the associated amount of material, can be neglected. This assumption is based on a theoretical consideration. A concentration of 1 ppm is already enough to provide full functionality and traceability of the modified polymer in the material used even after multiple processing steps (Figure 7 and Figure 8). Moreover, various studies [41,54,55,70,82] have confirmed the chosen concentration of modified polymers in different materials. Choosing higher concentrations of the modified polymer does not add value in terms of functionality or traceability [39]. The modified polymer is traceable in the initial material, as well as in the recycled material, down to a concentration of 1 ppm even after ten recycling steps (Figure 7 and Figure 8).

As there is no apparent correlation between the investigated concentration of the modified polymer and the determined mean value of the intensity of the main mass, only a qualitative analysis is available. This observation is confirmed by a previous study [39]. Additives in the injection molding material, like carbon black particles, are extracted from the injection molding material during the used extraction process (Table 5) and are deposited in the measuring chamber of the mass spectroscope during the MS/MS measurement [39]. Consequently, no precise quantification of the used modified polymer is possible. Although no quantification of the mean value of the intensity of the main mass is possible, the decrease in intensity recorded in recycling step R10, combined with the absence of a single sequence, could indicate a possible degradation of the modified polymer. Nevertheless, the functionality of the modified polymer is still present after ten recycling steps. Considering the manufacturing process of the used master batch, it can be expected that all granulates are marked and, thus, that there is sufficient dispersion of the modified polymer in the material [39].

Regarding the melting temperature, a small decrease occurred from R0 to R10 for all concentrations (Figure 2). This is in contrast to the observations of a previous study [29]. However, if only the change in melting temperature from R1 to R10 is considered, a small increase in melting temperature with increasing recycling steps can be observed. The melting temperature of the sample with the 30 ppm marker at R10 is considered to be an anomaly, based on the previous results. The increasing melting temperature is due to a higher crystallinity of the polymer via multiple processing. The reduction in molecular weight during recycling increases the mobility and folding ability of the chains, which results in the formation of thicker lamellae and, consequently, a higher degree of crystallinity [29]. However, crystallinity was not quantified in the investigation. The observed increase in the crystallization temperature from R0 to R10 (Figure 2) also resulted from the increasing degree of crystallinity and is confirmed by an earlier study [29]. It should be taken into account that the recorded changes in the respective melting and crystallization temperatures are based on a single measurement.

The investigated increasing mean value of the melt flow rate (MFR) from R0 to R10, illustrated in Figure 3, is in accordance with previous studies [18,19,29,83] that observed an increasing MFR with increasing numbers of process cycles. The observed small changes in the MFR up to R5 are in accordance with Aurrekoetxea et al. [29], who attributed this observation to the presence of stabilizers in the material. The MFR value reaches its maximum after the first recycling step. Further recycling no longer significantly shortens the mean chain lengths. The increasing MFR with increasing recycling steps is due to the decreasing molecular weight. Therefore, a decreasing melt viscosity of a polymer decreases with a decreasing molecular weight, and polymers with a decreasing molecular weight have an increasing melt flow rate [18,29,83,84]. A more robust statement would be provided via recording flow curves. Since the measurement is carried out below the relevant processing range, no direct conclusions can be drawn about the processing behavior [85,86].

The resulting investigated mechanical component properties at R0 are in accordance with previous studies [1,87]. With regard to Young’s modulus (Figure 4), there are different tendencies with increasing recycling steps, depending on the concentration of the marking agent. However, the changes were all within the respective standard deviation, and no clear tendency was observed. In contrast, previous studies have recorded both a decrease [18,20,84,88,89,90] and an increase [29,91] in Young’s modulus with increasing steps of recycling. For example, Tamrakar et al. [20] investigated a decrease in Young’s modulus of 12% after five recycling steps. Meneghetti et al. [88] indicated a decrease in Young’s modulus for polypropylene with increasing recycling content in the material by up to 20%. In contrast, Aurrekoetxea et al. [29] and Brostow and Corneliussen [91] attributed the increasing crystallinity in the material during recycling as the cause of the increasing Young’s modulus. The crystalline structures are much stiffer than amorphous structures and prevent rotations of the chain segments [29].

With regard to tensile strength (Figure 4), there are similar tendencies with increasing recycling steps, depending on the concentration of the marking agent. The decreasing tensile strength with recycling is in accordance with previous studies [18,20,84,88,90,92,93,94,95,96,97]. Multiple recycling steps lead to a change in material structure, which, due to degradation, results in a reduction in viscosity due to the scission of the chains and a significant loss of mechanical properties [18,84,90,92,93,94,95,96,97]. Tamrakar et al. [20] investigated a decrease in tensile strength of 4.6% after five recycling steps, and Meneghetti et al. [88] indicated a decrease in the tensile strength of polypropylene with increasing recycling content in the material by up to 20%. In contrast, Aurrekoetxea et al. [29] indicated increasing tensile strength with increasing numbers of recycling. Although it has not been investigated in this work, the decrease in tensile strength with increasing recycling steps could result from a thermomechanical degradation of the additives in the material during recycling [98].

Regarding elongation at break (Figure 4), there are different tendencies with increasing recycling steps, depending on the concentration of the marking agent. Previous studies [68,99] have mentioned that elongation at break exhibits a high statistical variation, which can be confirmed in the present study. The large spread of the results is probably due to a heterogeneous degradation in the material. Studies have shown that the oxidation of polyolefins (like polypropylene) is a heterogeneous process [5,100,101,102,103,104,105]. Due to the high standard deviations of the mean values, no discernible influence of either the recycling or the marking agent could be identified. Previous studies have recorded a decrease in elongation at break [5,18,29,83,84,89,106,107,108,109,110,111,112,113] with increasing steps of recycling. Decreasing elongation at break is due to the higher crystallinity of the recycled material [107] and to the reduction in the molecular weight with further recycling steps [29]. As a result of the reduced molecular weight, the density of bonding molecules incorporated into at least two crystalline lamellae, and also the number of those bonding the spherulites, decreases. In addition, the probability of chain entanglement in the amorphous phase decreases. As a result, the structure is less connected [106,109]. The concentration of binding molecules is also influenced by the crystallization temperature, as the density of the binding molecules decreases with increasing recycling due to shorter molecules and higher crystallization temperatures [29,106,110]. In addition, shorter molecules are less entangled and have fewer C-C bonds to stretch than long molecules of fresh material [29]. This assumption can be confirmed in this study (Figure 2).

Although no clear increase in Young’s modulus or tensile strength could be observed, as indicated by Aurrekoetxea et al. [29], the increasing melting and crystallization temperature indicate an increase in crystallinity. In contrast, the elongation at break decreases with increasing recycling steps, which could be partially confirmed in this work. The reason why the expected increases in Young’s modulus and tensile strength were not recorded in this study may be due to an insufficient number of recycling stages. The number of recycling steps was based on Aurrekoetxea et al. [29], who indicated an asymptotic behavior of the investigated mechanical properties after 10 recycling steps. Furthermore, even when the marking agent is recycled up to ten times, there are no marker-related degradation effects that result in an influence on the material and component properties. This is confirmed by the fact that the material and component properties at all recycling steps indicate no discernible influence of the marking agent. Even though the material properties of the investigated material and master batch differed slightly, and the component properties changed a little with the increase in the concentration of the modified polymer used, the variation of the investigated properties was within the standard deviation of the mean values.

Regarding Charpy impact strength (Figure 5), there are similar tendencies with increasing recycling steps, depending on the concentration of the marking agent. In contrast with earlier studies [83,88], Charpy impact strength increases with increasing recycling steps, increasing crystallinity (Figure 2) and increasing the melt flow rate (Figure 3). The result is, nevertheless, taken as a given since the complete breakage of the specimen was recorded in all Charpy impact tests [114]. One possible reason for the deviation from the previous study [83] is that a double-edge-notched specimen shape [65] was used. A possible explanation of why the marking agent had no discernible influence on Charpy impact strength is that polypropylene with a melt flow rate greater than 10 g/10 min (Table 6 and Figure 3) shows no change in notched impact strength with the addition of additives [83,115].

With regard to the Vicat softening temperature (VST) (Figure 6), there are similar tendencies with increasing recycling steps, depending on the concentration of the marking agent. Grellmann and Seidler [68] revealed that the softening behavior of polypropylene composites is mainly determined by the matrix. Compared to other studies [69,116], the VST is lower. This finding could be due to the elastomer-modified polypropylene compound used since the literature values refer to pure substances. Although the results of this study indicate a change in molecular weight due to recycling, this thermally induced damage to the material could not also be reflected in the VST [68,69]. Even though Arndt and Lechner [69] indicated the influence of additives on VST, this influence could not be confirmed in this study. This could be attributed to the reason that the chosen concentrations of the marking agent used were too low to influence the studied material and component properties using the selected testing methods [39].

## 5. Conclusions and Outlook

The identification and traceability of materials and components is a major challenge in the injection molding process. This challenge can be overcome through the use of a material-inherent marking technology. This allows the aging state of polymers and the composition of individual material mixtures to be monitored, as a result of which scatter-reducing process parameters can be set. Based on a systematic selection of modified polymers as a proper marking technology for application in polymer processing manufacturing, as well as the experimental validation of this marking agent in selective laser sintering in previous studies [38,39], this study focused on the investigation of the suitability of this marking agent in injection molding. In particular, the impact of the modified polymer used on the thermal and rheological material properties, as well as the mechanical and thermal component properties, during multiple processing steps of the marked material was investigated. In addition, the influence of multiple processing steps of the marked material on the presence and functionality of the used modified polymer was analyzed via mass spectroscopy. The key findings can be summarized as follows:

- Considering the applied investigations and concentrations of the modified polymer used in the injection molding material used, the marking technology does not have any discernible influence on the investigated properties of the material and components;
- During injection molding, the marked material shows analogous processing behavior to the unmarked material;
- The modified polymer used can be reliably detected in the material down to a concentration of 1 ppm, even after ten recycling steps;
- The concentration of the modified polymer and the measured intensity of the marking agent in the injection molding material used do not correlate;
- The dispersion of the modified polymer used in the injection molding material is sufficient;
- The modified polymer is suitable as a marking agent in injection molding;
- The modified polymer allows encoding of the material used at the molecular level.

The applicability of modified polymers for use in injection molding can be confirmed. This demonstrates the thermal stability of modified polymers [54,55] for application in the injection molding of polypropylene. Within the considered concentration ranges of the modified polymer, the marked injection molding material is substituted for the previously used injection molding material. While the modified polymer used has no identifiable impact on the investigated material and component properties, the modified polymer could conceivably be present in the material as a heterogeneous extraneous germ, influencing the crystallization and morphology of the polymer [117,118]. Moreover, it is possible that the modified polymer might become enriched in the amorphous phase of the polymer. This is because segregation occurs during crystallization, resulting in a segregation reaction [119]. An analysis of the injection molding material and master batch morphology could provide information about the possible effects of the modified polymer [13,15]. Furthermore, the use of a high-purity polymer could be useful to identify possible effects [39].

Based on this study’s findings, an injection-molding-specific coding strategy can be developed. The tracer-based process optimization [38] must be elaborated upon for the present application. This involves analyzing the materials at defined stages in the injection molding process and adjusting the process parameters in a targeted manner. For the implementation of the marking technology in injection molding, the extraction method has to be optimized. The avoidance of material-specific additives such as carbon black particles during MS/MS analysis could enable the quantification of modified polymers [39]. In addition, another method of extracting and transferring the modified polymers to the MS/MS device is conceivable. The application of the desorption electrospray-ionization MS/MS analysis method enables the possibility of measuring the marking agent in situ [56,120,121,122]. As a result, the traceability effort is minimized. In addition, the analytical equipment can be included in the injection molding process. Moreover, there are other potentially suitable marking technologies listed in an earlier study [38]. Their suitability for injection molding has to be evaluated as well.

## Figures and Tables

**Table 1:** Main molar mass and mass numbers of the sequences of the used modified polymers.

Main Molar Mass [Da]	Sequences [Da]
918.5	131.0|246.2|347.3|434.3|535.4|650.4|781.4|882.5|981.5

**Table 2:** Selected parameters of the injection molding machine used.

Option	Selected Setting
Temperatures (hopper to nozzle) [°C]	35|180|190|195|200|200
Mold temperature [°C]	40
Dosing quantity [cm^3^]	36.5
Decompression [cm^3^]	3
Screw speed [m/s]	0.25
Back pressure [bar]	80
Residual mass cushion [m^3^]	4.6
Volume flow [cm^3^/s]	16
Max. injection pressure [bar]	280
Switching volume [cm^3^]	7
Max. hold pressure [bar]	200
Hold time [s]	40
Residual cooling time [s]	18

**Table 3:** Breakdown of the test samples used for each process cycle.

Type	Dimension	Number	Usage
Tensile bar ^1^	1A ^2^	5	Tensile test
Tensile bar ^1^	1A ^2^	10	Vicat softening temperature test, Charpy impact test
Tensile bar ^1^	1A ^2^	2	Traceability

^1^ Tensile bars presented in Figure 1. ^2^ DIN EN ISO 527-2 [58].

**Table 4:** Selected parameters of the extrusion and pelleting machine used.

Option	Selected Setting
Temperature (hopper to nozzle) [°C]	50|190|190|190|190|190|195|195|200|200|200
Temperature at the output [°C]	209
Screw speed [1/min]	160
Nozzle head pressure [bar]	12
Volume flow [kg/h]	8
Capacity utilization [%]	30
Cut-off speed [m/min]	33

**Table 5:** Extraction methods used for the investigated samples [39].

Sample	Analyzed Quantity	Extraction Method
Granulate	5 g	Mixed with 10 mL of ethanol Placed in an ultrasonic bath at 40 °C for a duration of 30 min Filtration with a 22 µm filter Placed in a rotary vacuum evaporator Dilution with 2 mL of methanol and 3 mmol of ammonium acetate
Component ^1^		

^1^ Shredded pieces of tensile bar listed in Table 2 for analyzing traceability.

**Table 6:** Investigated material properties for the injection molding material and master batch used based on the injection molding material used and the modified polymer used.

Properties	Injection Molding Material	Master Batch
PE ^1^ onset crystallization temperature [°C]	113.16	113.09
PE crystallization temperature [°C]	110.02	109.95
PE onset melting temperature [°C]	107.86	107.01
PE melting temperature [°C]	121.31	120.95
PP ^2^ onset crystallization temperature [°C]	133.30	133.02
PP crystallization temperature [°C]	129.69	129.28
PP onset melting temperature [°C]	158.43	159.11
PP melting temperature [°C]	168.62	167.75
Max. pyrolytic degradation temperature [°C]	473.95	475.56
Residue [%]	9.1925	9.2435
Melt flow rate [g/10 min]	17.73 ± 0.19	17.31 ± 0.21

^1^ Polyethylene-specific peak. ^2^ Polypropylene-specific peak.

## Data Availability

The raw/processed data required to reproduce these findings cannot be shared at this time, as the data also form part of an ongoing study.

## References

[1] Isayev A.I., Kamal M.R., Liu S.-J. (2009). Injection Molding: Technology and Fundamentals.

[2] Yang Y., Gao F., Lu N., Chen X. (2016). Injection Molding Process Control, Monitoring, and Optimization.

[3] Rosato D.V. (2000). Injection Molding Handbook.

[4] Osswald T.A., Turng L.-S., Gramann P.J., Beaumont J. (2008). Injection Molding Handbook.

[5] Jansson A., Möller K., Gevert T. (2003). Degradation of post-consumer polypropylene materials exposed to simulated recycling—Mechanical properties. Polym. Degrad. Stab..

[6] Schyns Z.O.G., Shaver M.P. (2021). Mechanical Recycling of Packaging Plastics: A Review. Macromol. Rapid Commun..

[7] Lehner R., Weder C., Petri-Fink A., Rothen-Rutishauser B. (2019). Emergence of Nanoplastic in the Environment and Possible Impact on Human Health. Environ. Sci. Technol..

[8] Ölund G., Eriksson E. (1998). Resthandteringsalternativ för Plastförpackningar–en Miljöpåverknadsbedömmning.

[9] Yang Y., Boom R., Irion B., van Heerden D.-J., Kuiper P., Wit H.d. (2012). Recycling of composite materials. Chem. Eng. Process. Process Intensif..

[10] Gonçalves R.M., Martinho A., Oliveira J.P. (2022). Recycling of Reinforced Glass Fibers Waste: Current Status. Materials.

[11] Al-Salem S.M., Lettieri P., Baeyens J. (2009). Recycling and recovery routes of plastic solid waste (PSW): A review. Waste Manag..

[12] Ragaert K., Delva L., van Geem K. (2017). Mechanical and chemical recycling of solid plastic waste. Waste Manag..

[13] Ehrenstein G. (2011). Polymer-Werkstoffe: Struktur; Eigenschaften; Anwendung.

[14] Dahlmann R., Haberstroh E., Menges G. (2022). Menges Werkstoffkunde Kunststoffe, Vollständig neu Bearbeitete Auflage.

[15] Bonnet M. (2009). Kunststoffe in der Ingenieuranwendung: Verstehen und Zuverlässig Auswählen.

[16] Dostál J., Kašpárková V., Zatloukal M., Muras J., Šimek L. (2008). Influence of the repeated extrusion on the degradation of polyethylene. Structural changes in low density polyethylene. Eur. Polym. J..

[17] da Costa H.M., Ramos V.D., Rocha M.C. (2005). Rheological properties of polypropylene during multiple extrusion. Polym. Test..

[18] González-González V.A., Neira-Velázquez G., Angulo-Sánchez J.L. (1998). Polypropylene chain scissions and molecular weight changes in multiple extrusion. Polym. Degrad. Stab..

[19] Martins M.H., de Paoli M.-A. (2002). Polypropylene compounding with post-consumer material. Polym. Degrad. Stab..

[20] Tamrakar S., Couvreur R., Mielewski D., Gillespie J.W., Kiziltas A. (2023). Effects of recycling and hygrothermal environment on mechanical properties of thermoplastic composites. Polym. Degrad. Stab..

[21] Kaiser W. (2021). Kunststoffchemie für Ingenieure: Von der Synthese bis zur Anwendung.

[22] Abad M.J., Ares A., Barral L., Cano J., Díez F.J., García-Garabal S., López J., Ramírez C. (2004). Effects of a mixture of stabilizers on the structure and mechanical properties of polyethylene during reprocessing. J. Appl. Polym. Sci..

[23] Hamad K., Kaseem M., Deri F. (2011). Effect of recycling on rheological and mechanical properties of poly(lactic acid)/polystyrene polymer blend. J. Mater. Sci..

[24] Marrone M., La Mantia F.P. (1996). Re-stabilisation of recycled polypropylenes. Polym. Recycl..

[25] Catalina F. (1998). Degradación y estabilización de polipropileno. Rev. Plásticos Mod..

[26] Tiganis B.E., Shanks R.A., Long Y. (1996). Effects of processing on the microstructure, melting behavior, and equilibrium melting temperature of polypropylene. J. Appl. Polym. Sci..

[27] Ying Q., Zhao Y., Liu Y. (1991). A study of thermal oxidative and thermal mechanical degradation of polypropylene. Makromol. Chem..

[28] Hinsken H., Moss S., Pauquet J.-R., Zweifel H. (1991). Degradation of polyolefins during melt processing. Polym. Degrad. Stab..

[29] Aurrekoetxea J., Sarrionandia M.A., Urrutibeascoa I., Maspoch M.L. (2001). Effects of recycling on the microstructure and the mechanical properties of isotactic polypropylene. J. Mater. Sci..

[30] Chanda M., Roy S.K. (2016). Plastics Fabrication and Recycling.

[31] Marrone M., La Mantia F.P. (1996). Monopolymers blends of virgin and recycled polypropylene. Polym. Recycl..

[32] Steigemann U. (2012). Werkstoff- und ressourcenschonende Recyclingstrategien. Lightweight Des..

[33] Wenguang M., La Mantia F.P. (1996). Processing and mechanical properties of recycled PVC and of homopolymer blends with virgin PVC. J. Appl. Polym. Sci..

[34] Wenguang M.A., La Mantia F.P. (1995). Recycling of Post-consumer Polyethylene Greenhouse Films: Monopolymer Blends of recycled and virgin Polyethylene. Polym. Netw. Blends.

[35] Valenza A., La Mantia F.P. (1988). Recycling of polymer waste: Part II—Stress degraded polypropylene. Polym. Degrad. Stab..

[36] Pfaendner R., Herbst H., Hoffmann K., Sitek F. (1995). Recycling and restabilization of polymers for high quality applications. An Overview. Angew. Makromol. Chem..

[37] Auer M., Schmidt J., Diemert J., Gerhardt G., Renz M., Galler V., Woidasky J. (2023). Quality Aspects in the Compounding of Plastic Recyclate. Recycling.

[38] Eggers T., von Lacroix F., van de Kraan F., Reichler A.-K., Hürkamp A., Dröder K. (2023). Investigations for Material Tracing in Selective Laser Sintering: Part I: Methodical Selection of a Suitable Marking Agent. Materials.

[39] Eggers T., von Lacroix F., Goede M.F., Persch C., Berlin W., Dröder K. (2023). Investigations for Material Tracing in Selective Laser Sintering: Part II: Validation of Modified Polymers as Marking Agents. Materials.

[40] Lutz J.-F. (2015). Coding macromolecules: Inputting information in polymers using monomer-based alphabets. Macromolecules.

[41] Lutz J.-F. (2021). Les Polymères, Messagers à l’Échelle Moléculaire. IT Ind. Technol..

[42] Fearon P.K., Marshall N., Billingham N.C., Bigger S.W. (2001). Evaluation of the oxidative stability of multiextruded polypropylene as assessed by physicomechanical testing and simultaneous differential scanning calorimetry-chemiluminescence. J. Appl. Polym. Sci..

[43] Incarnato L., Scarfato P., Acierno D. (1999). Rheological and mechanical properties of recycled polypropylene. Polym. Eng. Sci..

[44] Incarnato L., Scarfato P., Gorrasi G., Vittoria V., Acierno D. (1999). Structural modifications induced by recycling of polypropylene. Polym. Eng. Sci..

[45] Kartalis C.N., Papaspyrides C.D., Pfaendner R., Hoffmann K., Herbst H. (1999). Mechanical recycling of postused high-density polyethylene crates using the restabilization technique. I. Influence of reprocessing. J. Appl. Polym. Sci..

[46] Dintcheva N., Jilov N., La Mantia F.P. (1997). Recycling of plastics from packaging. Polym. Degrad. Stab..

[47] Hopmann C., Michaeli W. (2017). Einführung in die Kunststoffverarbeitung.

[48] Moalli J. (2010). Plastics Failure: Analysis and Prevention.

[49] Eyerer P., Elsner P., Hirth T. (2008). Polymer Engineering: Technologien und Praxis.

[50] Baur E., Brinkmann S., Osswald T.A., Schmachtenberg E. (2013). Saechtling Kunststoff Taschenbuch.

[51] Domininghaus H., Elsner P., Eyerer P., Hirth T. (2012). Kunststoffe: Eigenschaften und Anwendungen.

[52] Pasquini N., Addeo A. (2005). Polypropylene Handbook.

[53] Gruden D. (2008). Umweltschutz in der Automobilindustrie: Motor, Kraftstoffe, Recycling.

[54] Dost G., Kummer B., Matloubi M., Moesslein J., Treick A. (2022). Produkt- und Materialpässe Nützen der Kreislaufwirtschaft Nur, Wenn sie Tatsächlich Robust mit Produkten und Materialien Verknüpft Sind! Kurzfassung, Freiburg, Germany. https://polysecure.eu/fileadmin/main/Unternehmen/Media-Files/220425_Produkt_Materialpass_Kurzfassung_Dt.pdf.

[55] Dost G., Matloubi M., Treick A., Kummer B. (2022). Booster für eine gelingende Kreislaufwirtschaft. Recycl. Mag. Sonderh.

[56] Youssef I., Carvin-Sergent I., Konishcheva E., Kebe S., Greff V., Karamessini D., Matloubi M., Ouahabi A.A., Moesslein J., Amalian J.-A. (2022). Covalent Attachment and Detachment by Reactive DESI of Sequence-Coded Polymer Taggants. Macromol. Rapid Commun..

[57] (2020). Kunststoffe—Polypropylen (PP)-Formmassen—Teil 2: Herstellung von Probekörpern und Bestimmung von Eigenschaften (ISO 19069-2:2016).

[58] (2012). Kunststoffe—Bestimmung der Zugeigenschaften—Teil 2: Prüfbedingungen für Form und Extrusionsmassen (ISO 527-2:2012).

[59] (2022). Kunststoffe—Thermogravimetrie (TG) von Polymeren—Teil 1: Allgemeine Grundsätze (ISO 11358-1:2022).

[60] Frick A., Stern C. (2017). Einführung in die Kunststoffprüfung: Prüfmethoden und Anwendungen.

[61] (2023). Kunststoffe—Dynamische Differenzkalorimetrie (DSC)—Teil 1: Allgemeine Grundlagen (ISO 11357-1:2023).

[62] (2012). Kunststoffe—Bestimmung der Schmelze-Massefließrate (MFR) und der Schmelze-Volumenfließrate (MVR) von Thermoplasten—Teil 1: Allgemeines Prüfverfahren (ISO 1133-1:2011).

[63] Mielicki C. (2014). Prozessnahes Qualitätsmanagement beim Lasersintern von Polyamid 12. Ph.D. Thesis.

[64] (2019). Kunststoffe—Bestimmung der Zugeigenschaften—Teil 1: Allgemeine Grundsätze (ISO 527-1:2019).

[65] (2010). Kunststoffe—Bestimmung der Charpy-Schlageigenschaften—Teil 1: Nicht Instrumentierte Schlagzähigkeitsprüfung (ISO 179-1:2010).

[66] (2014). Kunststoffe—Vielzweckprobekörper (ISO 3167:2014).

[67] (2023). Kunststoffe—Thermoplaste—Bestimmung der Vicat-Erweichungstemperatur (VST) (ISO 306:2022).

[68] Grellmann W., Seidler S. (2011). Kunststoffprüfung.

[69] Arndt K.-F., Lechner M.D. (2014). Polymer Solids and Polymer Melts–Mechanical and Thermomechanical Properties of Polymers.

[70] Al Ouahabi A., Amalian J.-A., Charles L., Lutz J.-F. (2017). Mass spectrometry sequencing of long digital polymers facilitated by programmed inter-byte fragmentation. Nat. Commun..

[71] Gunay U.S., Petit B.E., Karamessini D., Al Ouahabi A., Amalian J.-A., Chendo C., Bouquey M., Gigmes D., Charles L., Lutz J.-F. (2016). Chemoselective synthesis of uniform sequence-coded polyurethanes and their use as molecular tags. Chem.

[72] Lutz J.-F., Ouchi M., Liu D.R., Sawamoto M. (2013). Sequence-controlled polymers. Science.

[73] Guerrica-Echevarría G., Eguiazábal J.I., Nazábal J. (1996). Effects of reprocessing conditions on the properties of unfilled and talc-filled polypropylene. Polym. Degrad. Stab..

[74] Setnescu R., Barcutean C., Jipa S., Setnescu T., Negoiu M., Mihalcea I., Dumitru M., Zaharescu T. (2004). The effect of some thiosemicarbazide compounds on thermal oxidation of polypropylene. Polym. Degrad. Stab..

[75] Jipa S., Setnescu R., Setnescu T., Zaharescu T. (2000). Efficiency assessment of additives in thermal degradation of i-PP by chemiluminescence I. Triazines. Polym. Degrad. Stab..

[76] Gregorová A., Cibulková Z., Košíková B., Šimon P. (2005). Stabilization effect of lignin in polypropylene and recycled polypropylene. Polym. Degrad. Stab..

[77] Tocháček J. (2004). Effect of secondary structure on physical behaviour and performance of hindered phenolic antioxidants in polypropylene. Polym. Degrad. Stab..

[78] Gugumus F. (2000). Aspects of the impact of stabilizer mass on performance in polymers 2. Effect of increasing molecular mass of polymeric HALS in PP. Polym. Degrad. Stab..

[79] Chmela Š., Hrdlovič P. (1990). The influence of substituents on the photo-stabilizing efficiency of hindered amine stabilizers in polypropylene. Polym. Degrad. Stab..

[80] Hamid S.H. (2000). Handbook of Polymer Degradation.

[81] Gijsman P., Gitton M. (1999). Hindered amine stabilisers as long-term heat stabilisers for polypropylene. Polym. Degrad. Stab..

[82] Buttitta A. (2021). “Tracer-Based-Sorting”—Die Zukunft der Kunststoffverwertung. EU-Recycl. Fachmag. Eur. Recycl..

[83] Kausch H.-H. (2005). Intrinsic Molecular Mobility and Toughness of Polymers II.

[84] Canevarolo S.V. (2000). Chain scission distribution function for polypropylene degradation during multiple extrusions. Polym. Degrad. Stab..

[85] Wortberg J. (1996). Qualitätssicherung in der Kunststoffverarbeitung: Rohstoff-, Prozess- und Produktqualität; Tabellen.

[86] Pahl M., Gleißle W., Laun H.-M. (1995). Praktische Rheologie der Kunststoffe und Elastomere.

[87] (2007). World Encyclopedia. Modern Plastics Worldwide. https://www.plasticstoday.com/resin-pricing/.

[88] Meneghetti G., Ricotta M., Sanità M., Refosco D. (2016). Fully Reversed Axial Notch Fatigue Behaviour of Virgin and Recycled Polypropylene Compounds. Procedia Struct. Integr..

[89] Elloumi A., Pimbert S., Bourmaud A., Bradai C. (2010). Thermomechanical properties of virgin and recycled polypropylene impact copolymer/CaCO_3_ nanocomposites. Polym. Eng. Sci..

[90] Oblak P., Gonzalez-Gutierrez J., Zupančič B., Aulova A., Emri I. (2015). Processability and mechanical properties of extensively recycled high density polyethylene. Polym. Degrad. Stab..

[91] Brostow W., Corneliussen R.D. (1986). Failure of Plastics.

[92] Coulier L., Orbons H.G., Rijk R. (2007). Analytical protocol to study the food safety of (multiple-)recycled high-density polyethylene (HDPE) and polypropylene (PP) crates: Influence of recycling on the migration and formation of degradation products. Polym. Degrad. Stab..

[93] Scaffaro R., La Mantia F.P., Botta L., Morreale M., Tz. Dintcheva N., Mariani P. (2009). Competition between chain scission and branching formation in the processing of high-density polyethylene: Effect of processing parameters and of stabilizers. Polym. Eng. Sci..

[94] Mendes A.A., Cunha A.M., Bernardo C.A. (2011). Study of the degradation mechanisms of polyethylene during reprocessing. Polym. Degrad. Stab..

[95] Sun L., Zhao X.Y., Sun Z.Y. (2014). Study on the Properties of Multi-Extruded Recycled PE and PP. AMR.

[96] Yin S., Tuladhar R., Shi F., Shanks R.A., Combe M., Collister T. (2015). Mechanical reprocessing of polyolefin waste: A review. Polym. Eng. Sci..

[97] Baltes L., Costiuc L., Patachia S., Tierean M. (2019). Differential scanning calorimetry—A powerful tool for the determination of morphological features of the recycled polypropylene. J. Therm. Anal. Calorim..

[98] Gupta V.B., Mittal R.K., Sharma P.K., Mennig G., Wolters J. (1989). Some studies on glass fiber-reinforced polypropylene. Part II: Mechanical properties and their dependence on fiber length, interfacial adhesion, and fiber dispersion. Polym. Compos..

[99] Battisti M. (2015). Spritzgießcompoundieren von Polymer-Nanocomposites auf Basis von Schichtsilikaten. Ph.D. Thesis.

[100] Celina M., George G.A. (1993). A heterogeneous model for the thermal oxidation of solid polypropylene from chemiluminescence analysis. Polym. Degrad. Stab..

[101] Celina M., George G.A. (1995). Heterogeneous and homogeneous kinetic analyses of the thermal oxidation of polypropylene. Polym. Degrad. Stab..

[102] Gugumus F. (1998). Thermooxidative degradation of polyolefins in the solid state—6. Kinetics of thermal oxidation of polypropylene. Polym. Degrad. Stab..

[103] Gugumus F. (1998). Thermooxidative degradation of polyolefins in the solid state—7. Effect of sample thickness and heterogeneous oxidation kinetics for polypropylene. Polym. Degrad. Stab..

[104] Knight J.B., Calvert P.D., Billingham N.C. (1985). Localization of oxidation in polypropylene. Polymer.

[105] Richters P. (1970). Initiation Process in the Oxidation of Polypropylene. Macromolecules.

[106] Ibhadon A.O. (1998). Fracture mechanics of polypropylene: Effect of molecular characteristics, crystallization conditions, and annealing on morphology and impact performance. J. Appl. Polym. Sci..

[107] Greco R., Coppola F. (1986). Influence of crystallization conditions on the mechanical properties of isotactic polypropylene. Plast. Rubber Process. Appl..

[108] Seiffert S., Kummerlöwe C., Vennemann N. (2020). Makromolekulare Chemie: Ein Lehrbuch für Chemiker, Physiker, Materialwissenschaftler und Verfahrenstechniker.

[109] Karger-Kocsis J. (1995). Polypropylene Structure, Blends and Composites: Volume 3 Composites.

[110] Greco R., Ragosta G. (1988). Isotactic polypropylenes of different molecular characteristics: Influence of crystallization conditions and annealing on the fracture behaviour. J. Mater. Sci..

[111] Sugimoto M., Ishikawa M., Hatada K. (1995). Toughness of polypropylene. Polymer.

[112] Tjong S.C., Shen J.S., Li R. (1996). Morphological behaviour and instrumented dart impact properties of β-crystalline-phase polypropylene. Polymer.

[113] Jancar J., DiAnselmo A., DiBenedetto A.T., Kucera J. (1993). Failure mechanics in elastomer toughened polypropylene. Polymer.

[114] (2020). Kunststoffe—Bestimmung der Charpy-Schlageigenschaften—Teil 2: Instrumentierte Schlagzähigkeitsprüfung (ISO 179-2:2020).

[115] Moerl M. (2017). Steigerung der Zähigkeit von Isotaktischem Polypropylen durch Kontrolle der Morphologie Mittels 1,3,5-Benzoltrisamiden. Ph.D. Thesis.

[116] Carlowitz B. (1992). Tabellarische Übersicht über die Prüfung von Kunststoffen.

[117] Bastian M., Hochrein T. (2018). Einfärben von Kunststoffen: Produktanforderungen—Verfahrenstechnik—Prüfmethodik.

[118] Johnsen U., Spilgies G., Zachmann H.G. (1970). Abhängigkeit der heterogenen Keimbildung in Polypropylen von der Kristallisationstemperatur und von der Art der Beimengung der Fremdsubstanz. Kolloid Z.U.Z.Polym..

[119] Hornbogen E., Eggeler G., Werner E. (2017). Werkstoffe: Aufbau und Eigenschaften von Keramik-, Metall-, Polymer- und Verbundwerkstoffen.

[120] Amalian J.-A., Mondal T., Konishcheva E., Cavallo G., Petit B.E., Lutz J.-F., Charles L. (2021). Desorption electrospray ionization (DESI) of digital polymers: Direct tandem mass spectrometry decoding and imaging from materials surfaces. Adv. Mater. Technol..

[121] Takats Z., Wiseman J.M., Gologan B., Cooks R.G. (2004). Mass spectrometry sampling under ambient conditions with desorption electrospray ionization. Science.

[122] Cooks R.G., Ouyang Z., Takats Z., Wiseman J.M. (2006). Ambient mass spectrometry. Science.

